# Diagnosis and Medical Treatment

**Published:** 1951-10

**Authors:** John Apley


### THE DIAGNOSIS AND TREATMENT OF CONGENITAL HEART DISEASE*

BY

R. MILNES WALKER, M.S., F.R.C.S.
*Professor of Surgery in the University of Bristol*

AND

JOHN APLEY, M.D., M.R.C.P.
*Consultant Paediatrician, United Bristol Hospitals and Bath Royal United Hospital.*

### I—DIAGNOSIS AND MEDICAL TREATMENT

BY

JOHN APLEY

Is congenital heart disease rare or common? The question is well worth an answer before turning to the fascinating problems of diagnosis. Until recently it was accepted and taught that among children with organic heart disease only one case in ten was congenital in origin, with the remainder almost exclusively rheumatic. Recent surveys tell a very different story.

*Autopsy incidence.* News tends to grow less impressive with every mile it travels, and local information is always more interesting than that from a distance. Fortunately, therefore, autopsy figures are available from local sources. With Professor Hewer's permission I have examined the post-mortem records at the Department of Pathology, relating to deaths at the Bristol Royal Infirmary, General Hospital, Children's Hospital and Southmead Hospital, over the last ten years. It must be appreciated that they are concerned not with a general, but with a hospital population, and one, moreover, with an obstetric bias.

In 5,000 autopsies there were congenital anomalies of the heart in 108, roughly a proportion of one in fifty, or 2 per cent.

* Papers read to the Bristol Medico-Chirurgical Society, with X-ray films to illustrate the conditions, on Wednesday, 9th May, 1951.

In this total all live births have been included, but an appalling number of infants died within a short time of birth: one quarter (twenty-seven cases) did not live as long as one month, and more than half (sixty-seven cases) died within the first year. In a small proportion of cases the cardiac malformation was not the cause of death. In a survey of post-mortem evidence from world-wide sources, though apparently none has hitherto been available from this country, Brown (1950) also arrives at an average incidence of 2 per cent.: but he utters the caution that the inclusion of figures from children's hospitals and neo-natal departments tends to give an unduly high incidence.

*Cases at a cardiac clinic.* To clinicians, however, it is more appealing to contemplate patients than corpses, and here too local information is obtainable. It is natural to use rheumatic heart disease as a yardstick and I have had the welcome opportunity of taking part in an analysis of the attendances at Professor Perry's cardiac clinic for children, where ample material is available. At this clinic, over a period of six years (from 1943 to 1948), cases of congenital heart disease were slightly more than half as frequent as rheumatic heart cases. But when it is considered, as we have already seen, that many infants with congenital heart disease die before they are referred to any clinic, it becomes apparent that the proportions are more nearly equal This bears out the conclusion reached elsewhere (Keith, 1951), namely, that if all children from birth to school-leaving age are included the proportions of congenital and rheumatic heart disease are roughly the same.

These figures would appear extraordinary to previous generations of medical men, and one reason for the remarkable change in evaluation is that rheumatic heart disease is becoming distinctly less common than it was; another, which concerns us more closely here, is that congenital malformations of the heart are coming to be much more widely recognised.

*Relative incidence of cardiac malformations.* Like human figures, statistical ones are often dull and sometimes even repellent. To illustrate again our changing perspective I would, however, like to mention one last group of figures. Students are still taught what, until recently, clinicians believed: that simple pulmonary stenosis is one of the common congenital cardiac malformations. We now know that it is rare. In 300 consecutive children with congenital heart disease examined personally, many by the courtesy of my colleagues and especially Professor Perry, it was considered to be present in only 3 per cent. In 80 per cent. of all cases a definite diagnosis was made, and of these the six commonest malformations were as follows:

| | |
|---|---|
| Ventricular septal defect (VSD) . | 23 per cent. |
| Auricular septal defect (ASD) . | 15 ,, ,, |
| Patent ductus arteriosus (PDA) . | 15 ,, ,, |
| Tetralogy of Fallot . . . | 12 ,, ,, |
| Coarctation of the aorta . . | 6 ,, ,, |
| Transposition of great vessels . | 4 ,, ,, |

It should be noted that these proportions are affected by the addition of cases sent to the Surgical Unit at Bristol specifically for operation; at Bath, where this consideration does not apply, the figures for patent ductus and coarctation are less than half as high.

### Diagnosis

Since then we are dealing with a fairly common group of disorders, and one in which cure is sometimes possible, it must be accepted that diagnosis is of undoubted practical importance. The new and elaborate methods of investigation are producing results which are important and far-reaching, but these must eventually be re-stated in the language of clinical medicine. It is our task, as clinicians, to use them by giving to physical signs new values and new emphasis, and indeed this is already being achieved.

In nine patients of every ten with congenital heart disease an exact diagnosis can be made if all the ingenious tools of modern cardiology are employed. By combining the simple methods of clinical assessment, cardioscopy (visualising the heart by X-ray screening) and electrocardiography, an accurate diagnosis can be made in some three cases of every four, and these methods should certainly be used in hospital practice. By incorporating the new facts derived from these methods, clinical diagnosis itself is clearly advancing in precision. It would be over-optimistic to attempt to diagnose the more complicated malformations: instead, surely, the clinician should concentrate on the common anomalies. In the large majority of all cases a comprehensive diagnosis of congenital heart disease is possible, and in the six common malformations a presumptive diagnosis should be attainable in most instances at the bedside.

*Problems of Diagnosis.* The surgeon's problems are on a different plane from those of the physician. Where operation is contemplated it appears advisable, in all but the most straightforward cases (*e.g.* ligation of patent ductus arteriosus), to confirm the details of diagnosis and to plan operation with the help of preliminary investigations by some of the more elaborate methods, especially angiocardiography and cardiac catheterisation.

For the clinician's purposes, as compared with the surgeon's the problems are relatively straightforward.

(i) *Has the patient congenital heart disease?* The history can be helpful. It may suggest an inadequate blood supply, either systemic or pulmonary, which results in stunting of growth, poor exercise tolerance, breathlessness and the like. Or, especially in infancy, there may be evidence of cardiac or vascular pressure on thoracic viscera. Examination will be expected to reveal significant abnormalities, to be described shortly, and it is hardly necessary to stress that attention must not be confined to the heart alone.

(ii) *What is the type of malformation?* If there is *no* shunt, as in aortic or pulmonary stenosis or coarctation of the aorta, there may be symptoms and signs of diminished blood supply to the affected part, and possibly evidence of a compensatory collateral circulation. If there *is* a shunt, there may be evidence of deprivation of blood in one side of the circulation (systemic or pulmonary) and excess in the other side. In L-R shunt the most useful evidence is found in the lungs and in the right side of the heart. The classical sign of R-L shunt is cyanosis, but other findings are also helpful. L-R shunt, which includes such common anomalies as VSD, ASD and PDA, occurs more frequently than the reverse. Transposition of the great vessels in infants, and tetrology of Fallot in older patients, are the commonest malformations in which R–L shunt occurs.

(iii) *What is the site of the malformation?* This is determined largely from cardiac examination, mainly by the type of enlargement, alterations in valve sounds, and characteristics of any murmurs. But examination of the arteries and the viscera, especially the lungs, is also important.

*The history.* The infant may have stridor, bouts of dyspnoea, or evident attacks of pain on feeding. General appetite and development may be surprisingly good; but sometimes the child feeds with difficulty, or fails to thrive even if feeding is satisfactory. A history of repeated "lung infections" or "pulmonary congestion" is not infrequent. Some children have attacks suggestive of petit mal, in which they go pale and are dazed for a short time (Perry, 1931). The older child may tend to avoid strenuous games, become unduly breathless, or feel unwell after exertion. A few patients complain of what are called " growing pains ", presumably due to deficient blood supply to the legs, with such conditions as coarctation of the aorta or a wide ASD. The child may be permanently or transiently blue, the age of onset of cyanosis varying in different malformations; but it must be born in mind that most patients with congenital heart disease are not cyanosed.

*General examination.* In most cases, and especially after infancy, routine physical examination provides the earliest clue to the presence of congenital heart disease. The first abnormality detected is usually a cardiac murmur, though in diagnosis it is wiser to leave murmers to the last. The evidence from a murmur is always incomplete and may be misleading.

Growth may be impaired, both in cyanotic and non-cyanotic cases, but *failure to gain weight* is often more pronounced.

Cyanosis should be sought in the warm areas of the body, such as the mucous membrane of the mouth, since blueness of the extremities is often due to extra-cardiac causes. Central cyanosis is nearly always evidence not only of congenital heart disease but, more precisely, of a R-L shunt. It may be accentuated by crying, or by cold. In doubtful cases it should be looked for after exercising the patient. In the same circumstances undue breathlessness should be observed, and may be the only symptom to suggest a deficiency of blood to the lungs.

The chest wall merits careful attention, for chest deformities occur very frequently with congenital heart disease. The two conditions may be merely associated developmentally, but in some patients a characteristic deformity can be seen to develop *pari passu* with alterations in the shape and size of the heart.

In the lungs signs of congestion, if infection is absent, suggest the possibility of a L-R shunt. Moist sounds may be surprisingly sparse or absent with malformations, such as pulmonary stenosis, in which there is deficient blood supply to the lungs, even when pulmonary infection or cardiac failure supervenes.

Examination of the arteries may provide important information.

With one exception, the pulse pressure is normal or small in congenital heart disease: the exception is an extra-cardiac shunt. This is almost always caused by PDA, and the pulse pressure, though often normal, is never small and may be so great as to be " water-hammer " in type. A marked difference in the two radial pulses, due to unequal development, sometimes occurs with other cardiovascular anomalies. The femoral pulses should be carefully palpated in every child who is being examined, since their absence is evidence of coarctation of the aorta, a diagnosis which should also be considered if there is visible arterial pulsation above the manubrium sterni, or if the blood pressure in the arms is found to be high in children or adolescents.

### Cardiac Examination

*Cardiac enlargement* in infancy and early childhood is almost invariably due to congenital heart disease. If the apex beat is displaced downwards and to the left it is due to L ventricle enlargement (as in sub-aortic stenosis, and some cases of VSD or PDA); if outwards and to the left it is due to R ventricle enlargement (as in pulmonary stenosis and some cases of ASD). It is important, however, to detect R ventricle enlargement in those cases, too, such as tetralogy of Fallot, where the total heart size is not increased; when with R sided enlargement there has been corresponding underdevelopment of the L side. In these the apex beat is not likely to be displaced, but the sternum or ribs may be seen to protrude forward. In many cases the feel of the cardiac impulse is more helpful than its position: with R ventricle enlargement the cardiac impulse has a " flicking " or " tapping " character, quite different from the " thrust " or " heave " if the L ventricle is enlarged.

*Auscultation* of the heart sounds at the pulmonary area has become increasingly important in diagnosis. The second sound (P2) heard in this region is normally somewhat louder than the first sound during the early years of life, and is split into two elements. We are learning to recognise disturbances in the balance of the two sides of the circulation by their effects on the two elements of P2. In truncus arteriosus, pulmonary atresia, or other malformations where only a single large vessel is functioning, P2 is heard as a single and peculiarly sharp, almost " bell-like ", sound. If the pulmonary blood flow is reduced, as in the tetralogy of Fallot, the second element is diminished, and P2 is often soft. Where the pulmonary blood flow is greatly augmented, as in malformations with L-R shunt, the second element of P2 may be accentuated, or the two elements heard widely separated. With great accentuation of P2 the accompanying impulse may be readily palpable.

*Thrills and Murmurs.* The " classical " murmur of congenital heart disease is loud and harsh, is accompanied by a thrill, is systolic in time and maximal near the sternum or at the base of the heart. It is true that a murmur with these characteristics almost without exception signifies congenital heart disease, but often the murmur is by no means " classical ". With the largest defects there may be a soft murmur or no murmur. A thrill is not always detectable, especially in infancy and early childhood, and is not essential for the diagnosis. A diastolic (pulmonary or mitral) murmur is not uncommon, though nearly always it accompanies one in systole. The site of maximum intensity of a murmur may be difficult to determine, especially in babies; moreover it may vary in different individuals with identical malformations, or at different ages in any one patient.

The site of maximum intensity of the murmur is an approximate guide in locating the malformation. In infants it is usually impossible to define the site with accuracy, but in children and adults careful auscultation is helpful as long as it is borne in mind that in several types of malformation the murmur may be loudest at one and the same site.

### Common Malformations

The clinical picture with congenital malformations in infants and children may vary from that in adults, but it is often typical enough to permit a presumptive diagnosis. The commoner findings are briefly as follows:

*Ventricular septal defect.* The child is usually active and symptom-free, though a large, high defect may lead to congestive failure, especially in infants. The chest is not deformed and the pulses are normal or small. The heart is not enlarged. The second pulmonary sound is clearly split, often with accentuation of the second element. A loud, harsh, systolic murmur is heard, maximal in the third or fourth left interspace near the sternum. In older children it is usually accompanied by a thrill, the proportion of detected thrills increasing with age.

*Auricular septal defect.* The patient is thin and small, and the lower sternum protrudes, though in milder cases there are no such effects and no limitation of activity. The pulse pressure is small. The apex beat may be displaced outwards, and pulsation over the pulmonary area may be seen and felt. The cardiac impulse is " tapping " to the touch. The second pulmonary sound is widely split and often accentuated. A murmur, surprisingly often of only moderate intensity, is heard between the first and third left interspaces, close to the sternum. A thrill is unusual in early life, but becomes commoner later. A diastolic murmur may also be heard.

*Patent Ductus Arteriosus.* As a rule, the child is healthy and active, though in a few cases there is breathlessness on exertion or stunting of growth. The pulse pressure may be noteworthy in being wide. The cardiac impulse tends to be diffuse and heaving, and pulsation over the pulmonary area visible and palpable. The second pulmonary sound is loud and split. A harsh, continuous murmur is heard, rising in intensity during systole and fading in diastole. The murmur is usually widely audible but maximal over the pulmonary area, though it may, in a few cases and for some unknown reason, be heard only close to the sternum or under the clavicle. A thrill, either continuous or systolic, is almost always palpable if the typical murmur is present.

*Coarctation of the aorta.* A number of patients with this malformation, nearly always undiagnosed, die in infancy; those who survive are usually robust and active unless some additional anomaly is present. The diagnosis may first be entertained on hearing a systolic murmur over the precordium or at the back, on finding the blood-pressure to be raised, or on observing arterial pulsation in the neck; it is unusual in young children to see arterial pulsation in the chest wall, though it may be felt, especially if the patient leans forward with the arms hanging down. Coarctation of the aorta is confirmed by the absence of pulsation in the femoral arteries, and if this examination is regularly practised a number of cases will be found by this sign alone at an early age.

*Tetralogy of Fallot.* The child has usually been cyanosed from birth or soon after, but always within the first two years of life. He is nearly always thin, and usually under the average height for age. The pulses are normal or small. The sternum or the ribs to its left may protrude forward, though the heart is not clinically enlarged. The cardiac impulse is localised and tapping in character. The pulmonary second sound tends to be soft. Usually, a harsh systolic murmur is heard, which is maximal at the second or third left interspace, and often, especially in older children, accompanied by a thrill.

*Transposition of the Great Vessels.* The infant is cyanosed, usually from birth, but occasionally not for some weeks. There is failure to thrive, and often difficulty with feeding. There may be dyspnoea and inspiratory recession of the chest wall. In the lungs moist sounds are often heard. The heart is enlarged, and the second pulmonary sound accentuated. Murmurs are variable and may be completely absent, though usually a blowing systolic murmur is heard along the left sternal border.

### Medical Treatment

When we come to consider the enthralling subject of surgery in the treatment of cardiac malformations we do well to recall Osler's saying, that: "We stand too close to events to realise their tremendous significance." But obviously the physician, too, has an essential part to play in the management and treatment of the afflicted child.

It is important not to make a "cardiac invalid" of the patient. More often than not, as regards activities, the child adjusts himself to his disability better than the over-anxious doctor or parent does. In general, symptoms are a more reliable guide than are signs, though strenuous games should be barred if the heart is enlarged. A murmur, however loud and threatening it may sound, is not a valid reason for enforcing inactivity. The child should have hobbies and should be educated towards a sedentary occupation if there is a chance of his surviving into adult life.

Contrary to some older beliefs, cardiac failure in congenital heart disease is frequently recoverable, and should be treated with digitalis and the like. Paroxysmal dyspnoea is effectively treated with morphia. In the cyanosed child fluid should not be restricted, even for operations, for fear of inducing venous thrmoboses. Lastly, if the child has to have teeth or tonsils extracted, the physician may prevent the onset of bacterial endocarditis by prophylaxis with penicillin.

### REFERENCES

Brown, J. W. (1950).—*Congenital Heart Disease*, London, p.22.

Keith, J. D. (1951).—*Modern Trends in Paediatrics*, Ed. L. Parsons, London, 1951, p. 76.

Perry, C. B. (1931).—*Arch. Dis. Childhood*, **6**, 265.

### II—SURGICAL TREATMENT

BY

R. MILNES WALKER

